# Supplementary material for: Patterns of Intron Gain and Loss in Fungi
Source: PLoS Biol. 2004 Nov 30;2(12):e422. doi: 10.1371/journal.pbio.0020422 (PMC532390; doi:10.1371/journal.pbio.0020422)
Supplement: Table S1 — Also available at http://genes.mit.edu/NielsenEtAl/. (4.3 MB ZIP). [file pbio.0020422.st001.zip › NielsenEtAl/html/443.html]

AN1455.1.NCU02708.1.MG04773.1.FG00430.1


```
 CLUSTAL W (1.82) Multiple Sequence Alignments - Introns Inserted


Sequence 1: MG04773.1	664 aa
Sequence 2: FG00430.1	646 aa
Sequence 3: NCU02708.1	994 aa
Sequence 4: AN1455.1	719 aa
Alignment Length: 1005 aa
Number Identitical Residues: 414 aa
Alignment Score (without introns) 19880


MG04773.1 	-~-----------------------------------------------~----------
NCU02708.1	M0SSGKVKAGALWSKNKDELTKQLGELKTELGQLRIQKIVSSGTKLNKI2HDLRKSIARV
FG00430.1 	-~-----------------------------------------------~----------
AN1455.1  	-~-----------------------------------------------~----------
          	                                                            

MG04773.1 	------------------------------------------------------------
NCU02708.1	LTIINAKQRAQLRLFYKNKKYLPLDLRAKQTRAIRRRLSKEDASRVLEKTKKRTTHFPLR
FG00430.1 	------------------------------------------------------------
AN1455.1  	------------------------------------------------------------
          	                                                            

MG04773.1 	-----~------------------------------------------------------
NCU02708.1	KFAVK0VPPAVTYLSDVHVFCGSALPISVLFVDTPLSPHSWTLSIVVMSPVYSYNAAPTM
FG00430.1 	-----~------------------------------------------------------
AN1455.1  	-----~------------------------------------------------------
          	                                                            

MG04773.1 	----------------------------~-------------------------------
NCU02708.1	AKCAPTAVLQNIDDIECITPILPPYMLC1GGSSNCPLVAGALGIGLWLDLHQSVSDSEAL
FG00430.1 	----------------------------~-------------------------------
AN1455.1  	----------------------------~-------------------------------
          	                                                            

MG04773.1 	-----------------MSAASLSILGSKG--ESTRTLLRVIILFLIAGAAISSRLFSVI
NCU02708.1	QQPAPSRGRTNYQTTAIMSADSLERLASAGKGDSTRTLLRVIILLLVAGAAISSRLFSVI
FG00430.1 	---------------------------MTG--TNTRTLLRVLILFFIAGAAISSRLFSVI
AN1455.1  	-----------------MAESPVDVLLKGSSGRSARGLLRIIILVTIAAAAVSSRLFSVI
          	                  :  . .     ... .:* ***::**. :*.**:********

MG04773.1 	R1FESIIHEF1DPWFNFRATKYLVANGFYDFWDWFDDR~TWHPLGRVTGGTLYPGLMVTS
NCU02708.1	-~-------L1DPWFNFRATKYLVANGFYKFWDWFDDR~TWHPLGRVTGGTLYPGLMVTS
FG00430.1 	R1FESIIHEF1DPWFNFRATKYLVANGFYNFWDWFDDR~TWHPLGRVTGGTLYPGLMVTS
AN1455.1  	R1FESIIHEF1DPWFNFRATKYLVQNGFYDFWDWFDDR1TWHPLGRVTGGTLYPGLMVTS
          	   .:   .: ************* ****.******** *********************

MG04773.1 	GAIYHALRALTVPVDIRNVCVMLAPVFSGLTAFSAYLLTNEMTTSSSAGLLAAAFMGIAP
NCU02708.1	GVIYHALRALSIPVDIRNVCVLLAPAFSGLTAYAAYLLTNEMTPSSSAGLLAAIFMGIAP
FG00430.1 	GVIYHALRTLTVPVDIRNICVLLAPAFSGLTAYAAYLLTNEMTTTSSAGLLAAAFMGIAP
AN1455.1  	GVIYHILRFLTFPVDIRNICVLLAPGFSGLTAFAMYLLTNEMSSSPSAGLLAAAFMGIAP
          	*.*** ** *:.******:**:*** ******:: *******:.:.******* ******

MG04773.1 	GYISRSVAGSYDNEAIAIFLLVFTFYLWIKALKLGSMLWGALCALFYGYMVASWGG~YAF
NCU02708.1	GYISRSVAGSYDNEAIAIFLLVFTFYLWIKALKLGSMLWGALCALFYGYMVASWGG~YAF
FG00430.1 	GYISRSVAGSYDNEAIAIFLLVFTFYLWVKALKKGSMVIGGFCALSYGYMVASWGG~YAF
AN1455.1  	GYISRSVAGSYDNEAIAIFLLVFTFFLWIKAVKNGSIMWGALAALFYGYMVSAWGG2---
          	*************************:**:**:* **:: *.:.** *****::***    

MG04773.1 	ITCLLPLHSFVLICMGRFSTRLYISYTTWYALGTLASMQIPFVGFLPIKTSEHMPALG1I
NCU02708.1	ITCLLPLHAFVLICMGRYSTRLYVAYTTWYALGTLASMQIPFVGFLPVKTSEHMPALG1I
FG00430.1 	ITCLLPVHAFTLICMGRYSDRLYLSYTAWYVAGTLASFQIPFVGFLAVKTNEHMPALG1V
AN1455.1  	-------------------PRLYISYTTWYALGTLASMQIPFVGFLPIRNSDHMSALG1V
          	                    ***::**:**. *****:********.::..:**.*** :

MG04773.1 	FGFMQLMGFLDYVRSAIPGRQFRTFLVLFSAAIFVVGLSVLALATSAGYIAPWSGRFYSL
NCU02708.1	FGFLQLLAFLDYVRSAIPSRQFQTFLWVFAGSIFGIGLIGLVVATSAGFIAPWSGRFYSL
FG00430.1 	FGLLQVIAFILFVKRVIPGRQITTVVIAAGVATCMIGLAGLILLTSLGYIAPWGGRFYSL
AN1455.1  	FGLLQLVAFAEFVRSYIPGKQFQRLLTASIILITGLAFAGLVVLSMSGIIAPWSGRFYSL
          	**::*::.*  :*:  **.:*:  .:         :.:  * : :  * ****.******

MG04773.1 	WDTGYAKIHIPIIASVSEHQPTAWPAFFFDLNFLIWLFPAGVYLCFQNLRDEHVFIVVYA
NCU02708.1	WDTGYAKIHIPIIASVSEHQPTAWPAFFFDLNMLVWLLPAGVYLCYQKLADEHVFVIVYA
FG00430.1 	FDTGYAKVHIPIIASVSEHQPTAWPSFFFDMNFLIWLFPAGVYMCFQNLRDEHVFVVVYA
AN1455.1  	WDTGYAKIHIPIIASVSEHQPTAWPSFFFDLNFLIWLFPAGVYMCFRDLRDEHVFVIIYA
          	:******:*****************:****:*:*:**:*****:*::.* *****:::**

MG04773.1 	VFGSYFAGVMVRLMLTLTPVVCVAAAIAASSILDSFVSLKSPTPASDDADAKEAASKKAA
NCU02708.1	LFGSYFAGVMVRLMLTLTPVVCVAAAIALSTILHTYLKAKSPTPEELLPEESTDGTSKKA
FG00430.1 	MFASYFAGVMVRLILTLTPVVCVAAAIAASQLLETYMQIEQPDAAAETKSEGSESSKKN-
AN1455.1  	VLASYFAGVMVRLMLTLTPIVCVAAALVLSYILDTYLKT-SPGPVTRGRSNDESS-----
          	::.**********:*****:******:. * :*.:::.  .* .     .    .     

MG04773.1 	KEAAKGGLRSTNKPVVGVYTNLSKGLVTTAMTIYLLIFVLHCTWVTSNAYSSPSVVLASR
NCU02708.1	S---KSGLRASNKPYIGIYATFSKVTIVGLMTAYLLLFVAHCTWVTSNAYSSPSVVLASR
FG00430.1 	----KGALRALSKPSVGIYTVMSKVLVVGAMAIYLVMFVTHCTWVTSNAYSSPSVVLASR
AN1455.1  	-----EGLRSARSPEVGITSYFSKAVTTSAAIVYLLLFVAHCTWVTSNAYSSPSVVLASR
          	      .**:  .* :*: : :**   .     **::** ********************

MG04773.1 	LPDGSQHIIDDYREAYQWLRQNTKEDARIMSWWDYGYQIGGMADRPTLVDNNTWNNTHIA
NCU02708.1	MPDGSQHIIDDYREAYQWLRQNTKYDAKIMSWWDYGYQIGGMADRPTLVDNNTWNNTHIA
FG00430.1 	MPDGSQHIIDDYREAYQWLRQNTEKDDKVMSWWDYGYQIGGMADRPTLVDNNTWNNTHIA
AN1455.1  	MPDGSQFIIDDYREAYYWLRQNTPANAKIMSWWDYGYQIGGMADRPTLVDNNTWNNTHIA
          	:*****.********* ******  : ::*******************************

MG04773.1 	TVGKAMSSSEEVSYPIMRQHEVDYVLVVFGGLLGYSGDDINKFIWMVRIAEGIWPDEVKE
NCU02708.1	TVGKAMASREEVSYPIMRQHEVDYVLIVFGGLLGYSGDDINKFLWMVRIAEGIWPDEVKE
FG00430.1 	TVGKAMSSREEVSYPIMRQHEVNYVLVVFGGLIGYSGDDINKFLWMVRIAEGIWPDEVKE
AN1455.1  	TVGKAMSSREEVSYPILRQHDVDYVLVVFGGLLGYSGDDINKFLWMVRIAEGIWPDEVKE
          	******:* *******:***:*:***:*****:**********:****************

MG04773.1 	RDFFTNRGEYRVDDHATDTMKNSLM2YKM-------------------------------
NCU02708.1	REFFTPRGEYRVDAGATDTMKNSLM2YKMSYYNYNNLFPPGQAQDRVRGVRLPDEGPVLN
FG00430.1 	RNFFTARGEYKVNEEATETMKNSLM~----------------------------------
AN1455.1  	RDFFTARGEYRVDDQATPTMRNSLM2YKMSYHNFNKLFPMGQATDRVRGAKLPAESPQLS
          	*:*** ****:*:  ** **:****  . :  . ..  . ..: .   .   . ...  .

MG04773.1 	-------------------------------------------------------
NCU02708.1	TIEEAFTSENWIIRIYKVKDLDNLGRDHASASAFERGQKKKKVIKKKGPRVLRVD
FG00430.1 	-------------------------------------------------------
AN1455.1  	TLEEAFTSENWIIRIYKVKDLDNLGRDHSNAVAFDRGNKRKKASKRKGPRVLRSE
          	: ..: ::..      . .. .. . . :.: : . ... ..  . ...     .
```
